# Modulation of *MKNK2* alternative splicing by splice-switching oligonucleotides as a novel approach for glioblastoma treatment

**DOI:** 10.1093/nar/gky921

**Published:** 2018-10-17

**Authors:** Maxim Mogilevsky, Odelia Shimshon, Saran Kumar, Adi Mogilevsky, Eli Keshet, Eylon Yavin, Florian Heyd, Rotem Karni

**Affiliations:** 1 Department of Biochemistry and Molecular Biology, Institute for Medical Research Israel Canada, Faculty of Medicine, the Hebrew University of Jerusalem, 9112001 Jerusalem, Israel; 2 Institute for Drug Research, The School of Pharmacy, the Hebrew University of Jerusalem, 9112001 Jerusalem, Israel; 3 Department of Developmental Biology and Cancer Research, Faculty of Medicine, the Hebrew University of Jerusalem, 9112001 Jerusalem, Israel; 4 Institute of Chemistry and Biochemistry, Laboratory of RNA Biochemistry, Freie Universität Berlin, 14195 Berlin, Germany

## Abstract

The gene encoding the kinase Mnk2 (*MKNK2*) is alternatively spliced to produce two isoforms—Mnk2a and Mnk2b. We previously showed that Mnk2a is downregulated in several types of cancer and acts as a tumor suppressor by activation of the p38–MAPK stress pathway, inducing apoptosis. Moreover, Mnk2a overexpression suppressed Ras-induced transformation in culture and *in vivo*. In contrast, the Mnk2b isoform acts as a pro-oncogenic factor. In this study, we designed modified-RNA antisense oligonucleotides and screened for those that specifically induce a strong switch in alternative splicing of the *MKNK2* gene (splice switching oligonucleotides or SSOs), elevating the tumor suppressive isoform Mnk2a at the expense of the pro-oncogenic isoform Mnk2b. Induction of Mnk2a by SSOs in glioblastoma cells activated the p38–MAPK pathway, inhibited the oncogenic properties of the cells, re-sensitized the cells to chemotherapy and inhibited glioblastoma development *in vivo*. Moreover, inhibition of p38–MAPK partially rescued glioblastoma cells suggesting that most of the anti-oncogenic activity of the SSO is mediated by activation of this pathway. These results suggest that manipulation of *MKNK2* alternative splicing by SSOs is a novel approach to inhibit glioblastoma tumorigenesis.

## INTRODUCTION

The contribution of altered alternative splicing to cancer initiation and progression has become clear in recent years. Some genes encoding alternative splicing factors are mutated, amplified or deleted and act as oncogenes or tumor suppressors (1,2). The first alternative splicing factor shown to act as a proto–oncogene is a member of the SR family of proteins, SRSF1 (3,4). One of the important splicing targets of SRSF1 is *MKNK2*, encoding for the Mnk2 enzyme (5). The Mnk2 protein is one of two serine/threonine kinases (Mnk1, Mnk2) which are components of the Ras–MAPK pathway and were found to interact with and are phosphorylated by ERK1/2 and p38-MAPK. Mnk1/2 are the only known kinases responsible for phosphorylation of serine 209 of the eukaryotic translation initiation factor 4E (eIF4E), which binds the 5′ cap structure of mRNAs, and initiates cap-dependent translation (6). Enhanced mRNA translation initiation has been documented in many cancers. Phosphorylation of eIF4E by the Mnk proteins increases its affinity for capped mRNA, increases export and translation of several mRNAs implicated in tumorigenesis, and promotes the oncogenic activity of eIF4E (7,8).

**Figure 1.**
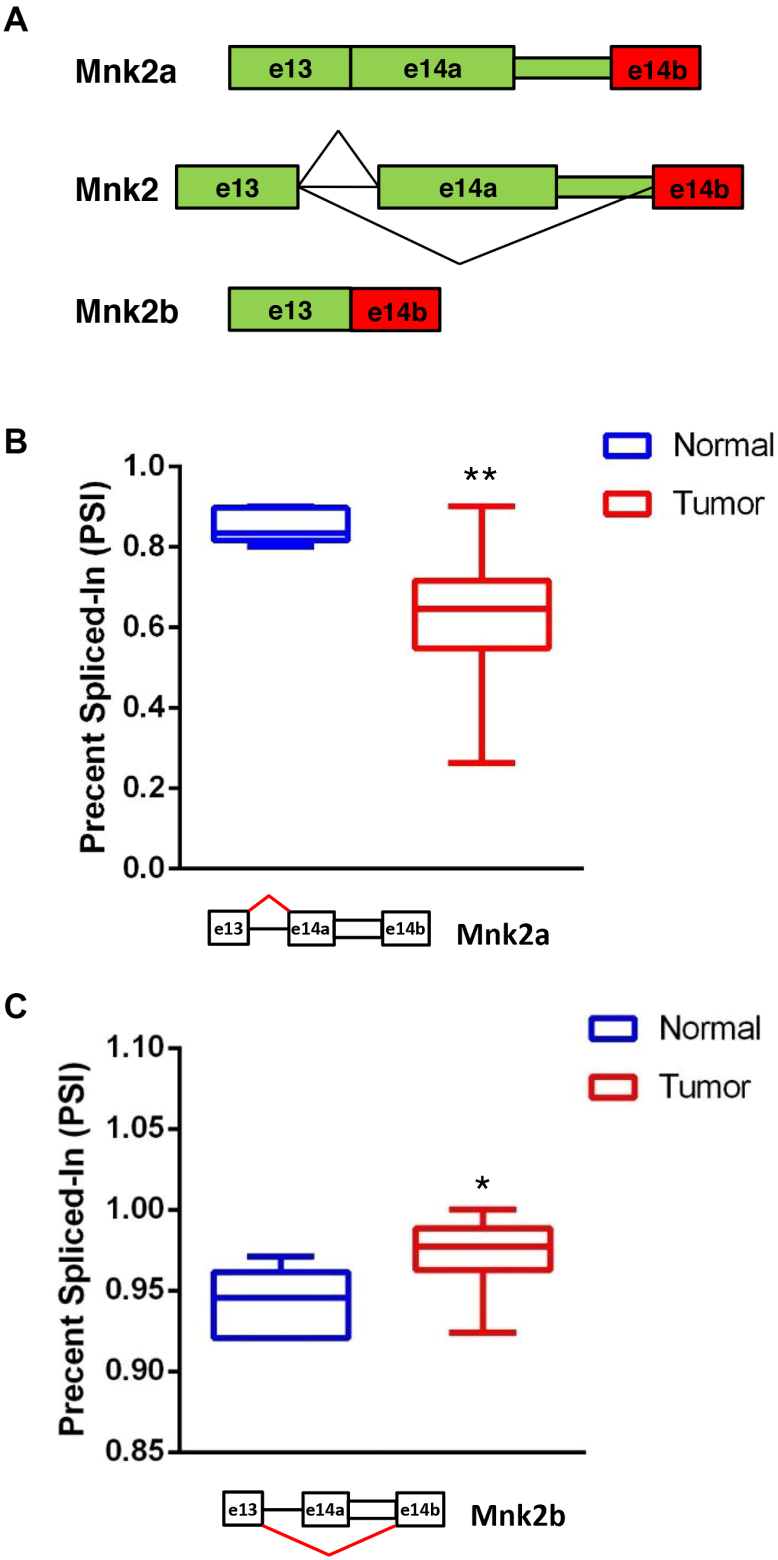
A *MKNK2* alternative splicing shift in glioblastoma. (**A**) Schematic representation of the alternative splicing of *MKNK2*. (**B**) Analysis of RNA-seq data from GBM and normal brain samples available at The Cancer Genome Atlas, shows lower PSI values for reads covering the junction between exon 13 and exon 14a (producing Mnk2a mRNA; indicated with red lines on the splicing scheme) and thus downregulation of Mnk2a in tumor samples compared to normal brain samples. (**C**) Analysis of RNA-seq data from GBM and normal brain samples available at The Cancer Genome Atlas, shows higher PSI values for reads covering the junction between exon 13 and exon 14b (producing Mnk2b mRNA; indicated with red lines on the splicing scheme) and thus upregulation of Mnk2b in tumor samples compared to normal brain samples. *P = 0.0017 two-sided, **P < 0.0001 two-sided, *n* = 5 for normal brain samples, *n* = 155 for tumor samples.

The human Mnk2 pre-mRNA undergoes alternative splicing which results in two proteins with different C-termini. The longer isoform of Mnk2, Mnk2a, possesses a MAPK binding site which is absent from the shorter isoform Mnk2b (Figure 1A) (9). The *MKNK2* pre-mRNA is a target of the splicing factor and oncoprotein SRSF1. Overexpression of SRSF1 reduces the levels of the Mnk2a isoform while increasing those of Mnk2b (3). Mnk2a was found to be downregulated in several cancers and acts as a tumor suppressor by co-localizing with, phosphorylating and activating p38–MAPK, inducing the transcriptional activation of its target genes and p38α–MAPK-mediated cell death. In addition, Mnk2a, but not Mnk2b, counteracts Ras-induced transformation both *in vitro* and *in vivo*, supporting its anti-oncogenic role (10). Even though overexpression of either Mnk2 isoform results in elevated levels of eIF4E phosphorylation, Mnk2b does not phosphorylate p38–MAPK, hence uncoupling eIF4E phosphorylation from the induction of the p38–MAPK stress response (10). Based on the findings that Mnk2a acts as a tumor suppressor and its production competes with the production of the oncogenic Mnk2b isoform, we focused our efforts on the manipulation of *MKNK2* alternative splicing to induce Mnk2a formation, in order to inhibit cancer cell growth and survival. We designed a set of Splice–Switching antisense oligonucleotides (SSOs), which bind to *MKNK2* pre-mRNA and screened for SSOs that interfere with selection of the Mnk2b splice site at the 3′ of *MKNK2* exon 14. We identified a SSO, 2b-block, which binds the junction between the Mnk2a UTR and exon 14b, making the site inaccessible to the splicing machinery, thus only allowing the use of the upstream 3′ splice site which generates Mnk2a. In this study we show that manipulation of *MKNK2* alternative splicing, elevating Mnk2a levels, inhibited survival and anchorage-independent growth of several cancer cell lines, sensitized glioblastoma cells to chemotherapy and inhibited glioblastoma tumor growth *in vivo*. Thus, our results suggest that *MKNK2* splicing modulation by SSOs can be developed as a novel therapy for glioblastoma.

## MATERIALS AND METHODS

### Cell culture

U87MG, HuH7 and MDA–MB–231 cell lines were grown in Dulbecco’s-modified Eagle’s medium (DMEM) supplemented with 10% (v/v) Fetal Bovine Serum (FBS), penicillin and streptomycin. U-251MG cell line was grown in Eagle's Minimum Essential Medium (EMEM) supplemented with 2 mM Glutamine, 1% non-essential amino acids, 1 mM Sodium Pyruvate and 10% (v/v) FBS, penicillin and streptomycin. In order to generate a U87MG cell line labeled with mCherry, cells were transduced with a recombinant retroviral vector expressing the mCherry fluorophore. Media was replaced 24 h post-transduction with selection media containing puromycin (2 μg/ml) for 72–96 h. The cells were then sorted using Flourescence-Activated Cell Sorter (FACS), in order to isolate the population with the strongest mCherry expression.

### Immunoblotting

Cells were lysed with Laemmli buffer and total protein concentration determined. A total 15 μg of total protein from each lysate was separated by sodium dodecyl sulphate-polyacrylamide gel electrophoresis and transferred onto a Polyvinylidene fluoride (PVDF) membrane (Invitrogen). The membranes were probed with the following primary antibodies: α-tubulin (1:2000 Sigma), total p38–MAPK (1:1000 Santa Cruz), phosphorylated p38–MAPK (1:1000 Cell Signaling). Secondary antibodies: HRP-conjugated goat anti-mouse and goat anti-rabbit IgG (H+L) (1:10 000, Jackson Laboratories).

### Intracranial injections

U87MG-mCherry cells were injected via stereotactic surgery into the murine striatum of NOD–SCID mice in both hemispheres, 2 mm lateral and to a depth of 3 mm to the bregma. Each hemisphere was injected with 2 μl of solution, comprised of 100 000 cells/μl resuspended in 12.5 μg/μl oligonucleotide solution. The mice were sacrificed after 2 weeks, the brains were extracted and visualized using Nikon SMZ18 stereomicroscope and NIS-Elements Br software. The brains were dissected and the tumors and surrounding tissue removed for RNA isolation. All animal experiments were performed in accordance with the guidelines of the Hebrew University committee for the use of animals for research.

### RT-PCR

Tissue samples were minced in TRI reagent (Sigma) using Next Advance Bullet Blender 24. Total RNA was isolated and cDNA was synthesized from 1 μg RNA using MMLV reverse transcriptase and oligo dT in a final volume of 25 μl according to the manufacturer's instructions (Promega). Polymerase chain reacion (PCR) was conducted on 1 μl of cDNA by KAPA 2G Fast HS ReadyMix PCR kit (KAPA Biosystems). PCR conditions were as described in manufacturer's protocol of ReadyMix with the addition of 5% (v/v) Dimethyl Sulfoxide (DMSO) for 35 cycles. PCR products were separated on 2% agarose gels or by LabChip GX/GXII Touch (PerkinElmer) (Primers: *MKNK2*, e12 For: gctgcgacctgtggagcctggg, e14a Rev: gatgggagggtcaggcgtggtc, e14b Rev: gaggaggaagtgactgtcccac; *GAPDH*, For: atcaagaaggtggtgaagcag, Rev: cttactccttggaggccatgt).

### qRT-PCR

Total RNA was extracted with TRI Reagent (Sigma), and 1 μg of total RNA was reverse transcribed using MMLV reverse transcriptase (Promega) after DNAse treatment (Promega). qPCR was performed on the cDNA using Fast SYBR Green Master Mix (Thermo Fisher Scientific) and CFX96 (Bio**-**Rad) real**-**time PCR machine (Primers: *c***–***FOS*, For: ctgtcaacgcgcaggactt, Rev: ggggctctggtctgcgat; *COX***–***2*, For: ccgaggtgtatgtatgagtgt, Rev: ctgtgtttggagtgggtttc; *IL***–***6*, For: caaattcggtacatcctcgac, Rev: gaaggttcaggttgttttctg; *β-actin*, For: ggcacccagcacaatgaaga, Rev: aggatggagccgccgatc).

### Oligonucleotides

The RNA oligonucleotides used in this study were synthesized with a full (all nucleotides) phosphorothioate (PS) backbone and each ribose 2′-hydroxyl is modified to either 2′-*O*-methyl (2′-OMe) or 2′-methoxyethyl (2′-MOE) as specified in the figure legends. PS greatly increases the stability and deliverability of the oligos which is further improved by the 2′ modifications (11). SCR: ucacuuccuccucccucccc; 2b–block: ggaagugacugucccaccuucaga; 2a-block: cagcuguuccugggaaacgggg; +1: ggagggaggaggaagugacu; +2: acagggguggggagggagga; +3: cguggaugcgacaggggugg. In 2′-MOE SSOs thymidine was incorporated instead of uridine.

### Transfection of oligonucleotides

Cells were transfected with oligos using lipofectamine 2000 (Invitrogen) according to the manufacturer's protocol.

### Clonogenic assay

Twenty-four hours post-transfection 1000, 500 and 250 cells were seeded in duplicates in 6-well plates with 2 ml of media (DMEM, 10% FBS). After 10–21 days cells were fixated with 2.5% glutaraldehyde solution for 10 min, stained with 1% methylene blue solution, photographed and counted.

### Anchorage–independent growth

Twenty-four hours post-transfection 15 000 cells per well were seeded in duplicates in 6-well plates. Each well was coated with 2 ml of bottom agar mixture (media, 10% FBS, 1% agar). After the bottom layer had solidified, 2 ml of top agar mixture (media, 10% FBS, 0.3% agar) containing the cells was added. After this layer had solidified, 2 ml of media (media, 10% FBS) was added. Plates were incubated at 37 °C with 5% carbon dioxide. Following 10–21 days, colonies from 10 different fields were counted and the average number of colonies per well was calculated.

### Trypan blue exclusion assay

Twenty-four hours post-transfection 50 000 cells per well were seeded in triplicates in 12-well plates. The following day media was replaced with media containing either 8 μg/ml of doxorubicin, 50–100 μM of CDDP, 500 μM of temozolomide (TMZ) or DMSO control. After 48 h for doxorubicin and CDDP and 72 h for TMZ cells were trypsinized and spun down, media and phosphate-buffered saline (PBS) wash were also collected. Cells were resuspended in HBSS and the percentage of dead cells was determined using 0.4% trypan blue and a BioRad cell counter.

### Statistics

Mann–Whitney *U*-test was used for the analysis of the RNA-seq data from TCGA. Two tailed Student's *t*-test was used for the analysis of all the other experiments.

## RESULTS

### A switch in *MKNK2* alternative splicing occurs in GBM

*MKNK2* is alternatively spliced to yield two isoforms: Mnk2a and Mnk2b (9). We have shown previously that Mnk2a is downregulated in several cancer types and acts as a tumor suppressor by co-localizing with, phosphorylating and activating p38α–MAPK, inducing the transcriptional activation of its target genes and p38α–MAPK-mediated cell death (10). Due to the unknown role of *MKNK2* alternative splicing in glioblastoma development and progression, we sought to examine the ratio of *MKNK2* splice isoforms in glioblastoma patient-derived samples. We mined the RNA-seq data from normal brain and glioblastoma multiforme (GBM) samples available from The Cancer Genome Atlas (TCGA; https://cancergenome.nih.gov). We used the SpliceSeq resource (12) to analyze RNA-seq data of the reads covering the junction between exon 13 and either exon 14a (producing Mnk2a mRNA) or exon 14b (producing Mnk2b mRNA). We observed lower PSI (percent spliced in; denotes the fraction of mRNAs that represent the inclusion isoform (13)) for the junction between exon 13 and exon 14a (Mnk2a; Figure 1B) and higher PSI for the junction between exon 13 and exon 14b (Mnk2b; Figure 1C) in tumors as compared to normal brain. These results suggested that, similar to what we found in other cancers (10), Mnk2a levels are downregulated while Mnk2b levels are upregulated in glioblastoma. In light of the fact that Mnk2a acts as a tumor suppressor and is downregulated in several cancers, including glioblastoma, we hypothesized that manipulating *MKNK2* splicing to elevate Mnk2a and reduce Mnk2b will inhibit glioblastoma development and progression.

### The 2b-block SSO switches *MKNK2* alternative splicing in a dose-dependent manner

**Figure 2.**
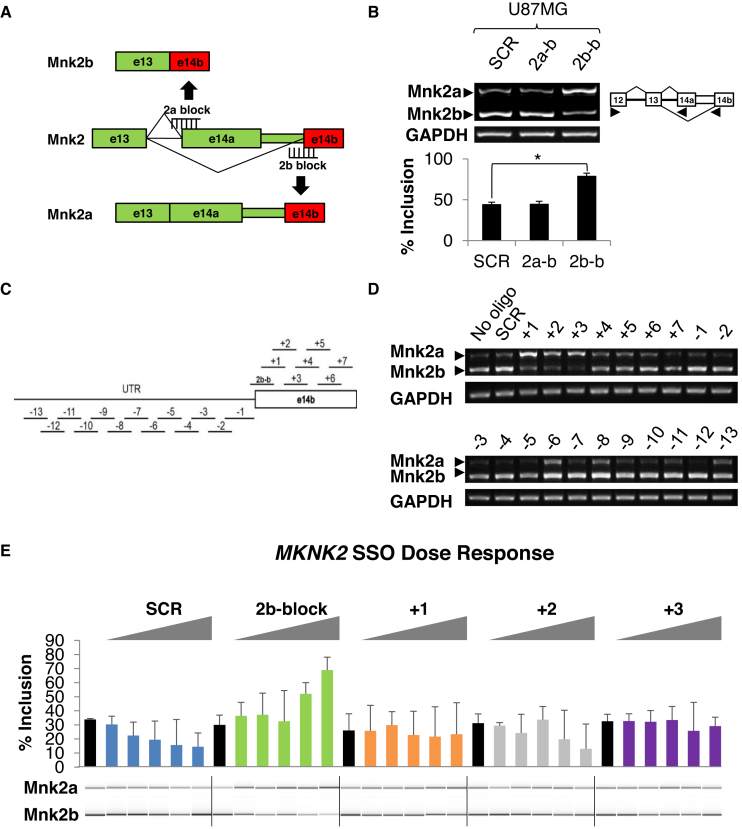
The 2b-block SSO switches *MKNK2* alternative splicing in a dose-dependent manner. (**A**) Schematic representation of the steric blocking SSOs and their binding sites. The 2b-block SSO binds the splice junction between the Mnk2a UTR and exon 14b, blocking access of the splicing machinery, producing the tumor suppressive isoform Mnk2a. The 2a-block SSO binds the splice junction between intron 13 and exon 14a, promoting the formation of Mnk2b. ( **B**) RT-PCR analysis of mRNA isolated from U87MG glioblastoma cells transfected with 2.5 μM of either SCR, 2a-block or 2b-block 2′-OMe oligos 72 h post-transfection. *P < 0.01, *n* = 6 for each group. (**C**) Schematic representation of the oligonucloetide walk. The SSOs and their binding sites are shown. The 2b-block SSO binds the splice junction between the Mnk2a UTR and exon 14b. The (+) SSOs bind along the 14b exon, with 10 nt overlap. The (−) SSOs bind along the region upstream of the 3′ splice site of exon 14b, with 5 nt overlap. (**D**) RT-PCR analysis of mRNA isolated from U87MG glioblastoma cells transfected with 5 μM of the indicated 2′-OMe SSOs 72 h post-transfection. (**E**) LabChip quantitation (and representative picture) of RT-PCR analysis of mRNA isolated from U87MG glioblastoma cells transfected with the indicated 2′-MOE (PS backbone) SSOs at the concentrations of 100 nM, 200 nM, 500 nM, 1 μM and 2.5 μM, 72 h post-transfection. Black bars for cells without oligo. *n* = 3 for each sample.

In an attempt to modulate *MKNK2* splicing, we designed 2′-OMe SSOs to mask the 3′ splice sites of the alternative exons 14a or 14b of *MKNK2*, which would result in a shift in the ratio between Mnk2a and Mnk2b mRNA isoforms. The 2a-block SSO was designed to promote production of the Mnk2b isoform and the 2b-block SSO was designed to upregulate Mnk2a (Figure 2A). U87MG glioblastoma cells, consistent with our TCGA analysis, express low levels of Mnk2a mRNA, making them an appropriate system for manipulation of the splicing event to elevate the tumor suppressive isoform levels (Figure 2B). Hepatocellular carcinoma cells HuH7 and metastatic breast carcinoma cells MDA–MB–231 also express low levels of Mnk2a mRNA (Supplementary Figure S1A). We observed that the 2b-block SSO efficiently upregulated the Mnk2a isoform mRNA in all three cell lines when compared to cells treated with the scrambled sequence (SCR; Figure 2B and Supplementary Figure S1A). Following the observation that the 2b-block SSO upregulates the Mnk2a isoform, we wanted to determine if we could pinpoint a more effective SSO in this region. To this end we performed an oligonucleotide walk, for which we designed and synthesized 20 overlapping non-PS 2′-OMe SSOs; 13 targeting the region upstream of the 3′ splice site of exon 14b and 7 along exon 14b (Figure 2C). Each SSO was individually transfected into U87MG cells. Several SSOs appeared to influence *MKNK2* alternative splicing, with the +1, +2 and +3 SSOs (in exon 14b) considerably upregulating Mnk2a levels and downregulating Mnk2b levels (Figure 2D). The 2b-block, +1, +2, +3 SSOs and SCR oligo were resynthesized, on a small scale, incorporating a currently clinically relevant 2′-MOE modification (11). These newly synthesized SSOs were transfected into U87MG cells at a range of concentrations (100 nM–2.5 μM). All the SSOs (+1, +2, +3 and 2b-block) showed upregulation of Mnk2a levels in a dose-dependent manner (Figure 2E). However, the 2b-block SSO was clearly superior, showing greater effect on *MKNK2* splicing, elevating Mnk2a levels while reducing Mnk2b. Taken together these results show that multiple SSOs cause upregulation of Mnk2a isoform, with the 2b-block SSO being the most efficient. The fact that this effect is dose responsive and not all the SSOs behaved similarly (including SCR sequences) reinforces the conclusion that the effect is specific. Transfection of the 2′-MOE SCR and 2b-block oligos into another glioblastoma cell line, U251MG, showed similar results. 2b-block treated cells showed a substantial upregulation of Mnk2a, strengthening the conclusion of the effect of the SSOs (Supplementary Figure S2A). It should be noted that the 2′-MOE oligos were transfected at a lower concentration (1 μM) given their improved cellular penetration.

### The 2b-block SSO activates the p38α–MAPK stress pathway and inhibits the oncogenic properties of glioblastoma, breast and HCC cancer cells in culture

**Figure 3.**
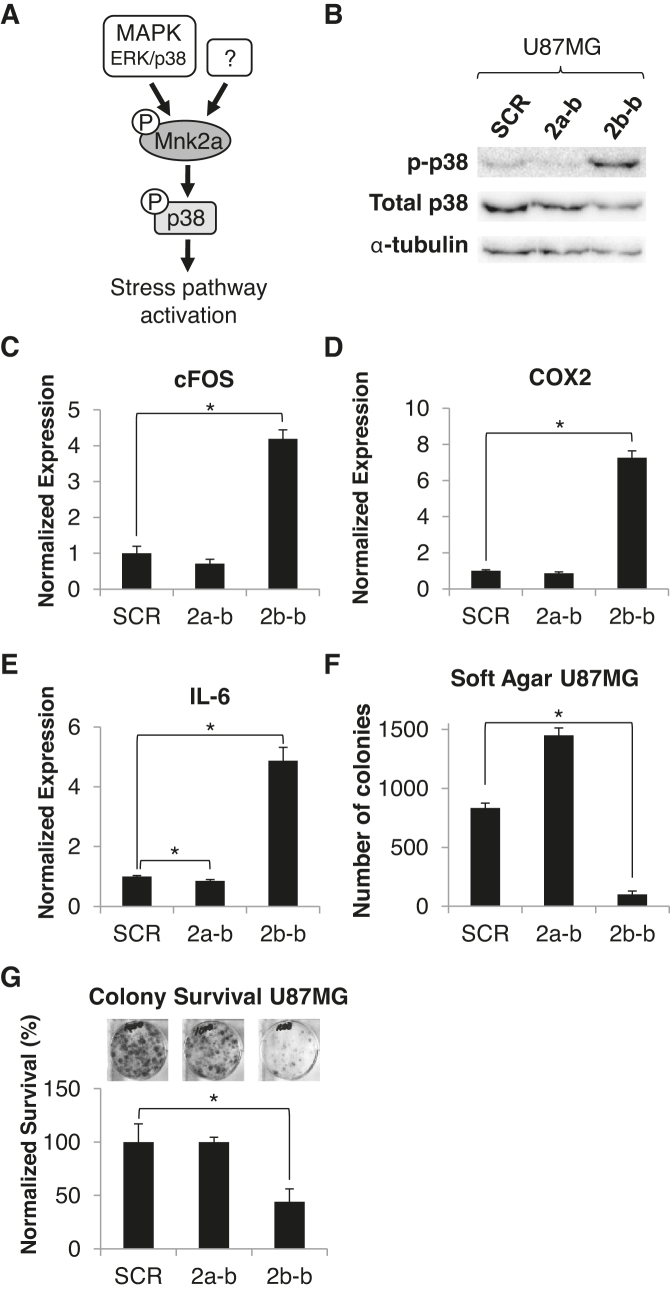
The 2b-block SSO activates the p38α–MAPK stress pathway and inhibits the oncogenic properties of glioblastoma cells in culture. (**A**) A scheme showing that Mnk2a is a substrate of p38. Cancer cells express low levels of Mnk2a, which is capable of phosphorylating and activating p38 inducing the transcriptional activation of its target genes and p38-mediated cell death. (**B**) Western blot analysis of protein lysates from U87MG glioblastoma cells transfected with 2.5 μM of either SCR, 2a-block or 2b-block 2′-OMe oligos 72 h post-transfection. Membranes were probed with the indicated antibodies. (**C–E**) qRT-PCR analysis of mRNA isolated from U87MG glioblastoma cells transfected with 2.5 μM of either SCR, 2a-block or 2b-block 2′-OMe oligos 72 h post-transfection. *P < 0.01 two-sided, *n* = 3 for each group. (**F**) Colony formation in soft agar assay performed on U87MG glioblastoma cells transfected with 2.5 μM of the indicated 2′-OMe oligos. *P < 0.01 two-sided, *n* = 3 wells for each group, in each well 10 fields were counted. (**G**) Clonogenic assay performed on U87MG glioblastoma cells transfected with 2.5 μM of the indicated 2′-OMe oligos and seeded at low density. The number of colonies in each treatment was normalized to number of colonies in cells treated with SCR oligo. *n* = 3 wells for each group.

We have previously shown that Mnk2a acts as a tumor suppressor through phosphorylation and activation of p38α–MAPK, leading to the transcriptional activation of its target genes (10) (Figure 3A). Consistent with this, SSO-mediated promotion of Mnk2a production significantly increased the phosphorylation of p38α–MAPK (Figure 3B; Supplementary Figures S1B and 2B) and the mRNA levels of its target genes (Figure 3C–E; Supplementary Figures S1C-D and 2C). Moreover, in some cases we observed a reduction in p38α–MAPK phosphorylation and target genes’ mRNA levels following transfection with the 2a-block SSO which promotes Mnk2b production (Figure 3B–E; Supplementary Figure S1B–D), further corroborating the relationship of *MKNK2* alternative splicing with p38α–MAPK activation. These results suggest that modulation of *MKNK2* alternative splicing by an SSO, which elevates Mnk2a levels, activates the p38–MAPK stress pathway.

**Figure 4.**
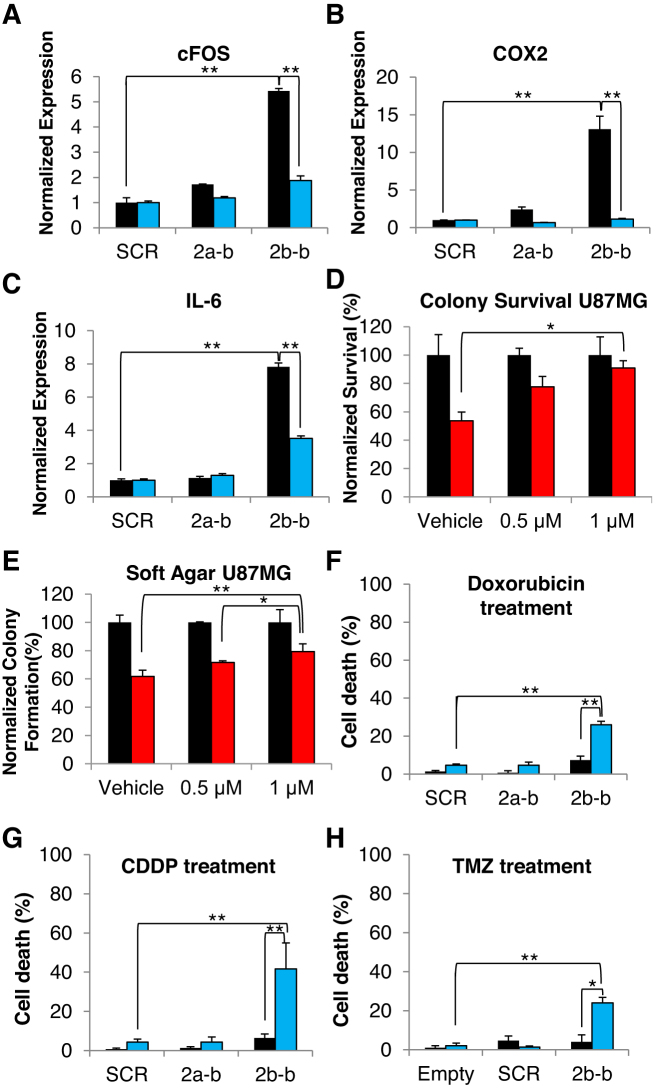
The cytotoxic activity of the 2b-block SSO is p38-MAPK dependent and it can sensitize glioblastoma cells to chemotherapeutic drugs in culture. (**A–C**) qRT-PCR analysis of mRNA isolated from U87MG glioma cells, transfected with 2.5 μM of either SCR, 2a-block or 2b-block 2′-OMe oligos and treated with SB203580 (blue bars) or no treatment (black bars) for 72 h post-transfection. *n* = 3 for each group. (**D**) Clonogenic assay performed on cells transfected with 2.5 μM of either SCR (black) or 2b-block (red) 2′-OMe oligos and treated with either vehicle, 0.5 or 1 μM of SB203580 for the duration of the experiment. The number of colonies in each treatment was normalized to the number of colonies in cells treated with SCR oligo. *n* = 2 wells for each group. (**E**) Colony formation in soft agar assay performed on cells transfected with 2.5 μM of either SCR (black) or 2b-block (red) 2′-OMe oligos and treated with either vehicle, 0.5 or 1 μM of SB203580 for the duration of the experiment. The number of colonies in each treatment was normalized to number of colonies in cells treated with SCR oligo. *n* = 2 wells for each group, in each well 10 fields were counted. *P < 0.05 two-sided. **P < 0.01 two-sided. (**F**) Trypan blue exclusion assay performed on U87MG cells transfected with 2.5 μM of the indicated 2′-OMe oligos and treated with 8 μg/ml doxorubicin (blue bars) or vehicle (black bars) for 48 h. **P ≤ 0.01 two-sided, *n* = 3 wells for each group. (**G**) Trypan blue exclusion assay performed on U87MG cells transfected with 2.5 vμM of the indicated 2′-OMe oligos and treated with 50 μM CDDP (blue bars) or vehicle (black bars) for 48 h. *P ≤ 0.05 two-sided, **P ≤ 0.01 two-sided, *n* = 3 wells for each group. (**H**) Trypan blue exclusion assay performed on U87MG cells transfected with 1 μM of the indicated 2′-MOE oligos and treated with 500 μM TMZ (blue bars) or vehicle (black bars) for 72 h. *P ≤ 0.05 two-sided, **P ≤ 0.01 two-sided, *n* = 3 wells for each group.

Overexpression of the Mnk2a isoform in Ras-transformed MCF–10A cells inhibits anchorage-independent growth, survival and tumor formation *in vivo* (10). Consistent with these findings, we observe impaired anchorage independent growth in U87MG and U251MG cells transfected with the 2b-block SSO, as analyzed by colony formation in soft agar (Figure 3F and Supplementary Figure S2D). The anchorage independent growth of MDA–MB–231 cells was also significantly damaged by the 2b-block SSO (Supplementary Figure S3A). In addition, the 2b-block SSO restricted survival and proliferation of U87MG and U251MG cells, as analyzed by a clonogenic assay (Figure 3G and Supplementary Figure S2E). HuH7 and MDA–MB–231 cells exhibited similar inhibition of survival following transfection of 2b-block SSO (Supplementary Figure S3B and C). This effect, decreased anchorage independent growth and survival, was also observed in U87MG cells transfected with the 2′-MOE 2b-block SSO when compared to the 2′-MOE SCR oligo. The 2′-MOE SSO consistently showed the same effect as the 2′-OMe modified SSO, despite the lower concentrations (500 nM and 1 μM) (Supplementary Figure S3D and E). Next, we wanted to determine whether inhibition of the oncogenic potential in cells transfected with 2b-block SSO, is mediated through the activation of the p38–MAPK pathway due to elevated levels of Mnk2a. To this end, we inhibited p38–MAPK activity in U87MG cells transfected with the SSOs and repeated the clonogenic and soft agar assays in the absence and presence of a specific p38–MAPK inhibitor SB203580 (10,14,15). SB203580 does not perturb the phosphorylation of p38α–MAPK but inhibits its downstream activity, including the ability to induce transcription of its target genes. Indeed, we observed that in U87MG cells treated with SB203580, the mRNA levels of the p38α–MAPK target genes induced by the 2b-block SSO, were reduced to levels similar to those in the SCR treated samples (Figure 4A–C). Similarly, the reduction in survival and anchorage independent growth in the 2b-block SSO treated cells, relative to SCR treated cells, was reversed upon inhibition of p38α–MAPK with SB203580 (Figure 4D and E). Altogether, these results suggest that the 2b-block SSO inhibits the oncogenic potential of glioblastoma cells by activation of the p38α–MAPK stress pathway.

### Induction of Mnk2a by the 2b-block SSO re-sensitized glioblastoma cells to chemotherapy

Drug resistance of cancer cells is a major roadblock in cancer treatment, specifically for glioblastoma treatment (16). Aberrant pre-mRNA splicing can promote cell survival in response to chemotherapy by deregulation of apoptosis, drug metabolism or altering the sequence of drug targets (17). Given the fact that the longer isoform, Mnk2a, activates the p38–MAPK stress response, which inhibits cell survival and promotes apoptosis, we hypothesized that shifting the alternative splicing of *MKNK2* toward more Mnk2a could re-sensitize drug resistant tumors to chemotherapy. To test this hypothesis we transfected U87MG cells with SSOs and treated the cells with either doxorubicin, cisplatinum (CDDP) or TMZ, three known chemotherapeutic agents. We found that while either chemotherapeutic agent alone or the 2b-block SSO alone had little effect on cell death, cells transfected with the 2b-block SSO combined with chemotherapeutic treatment showed an enhanced effect. We detected ∼6-fold increase in cell death following doxorubicin treatment, ∼8-fold increase in cell death following CDDP treatment and approximately 6–fold increase in cell death following TMZ treatment in 2b-block SSO transfected cells compared to SCR or 2a-block SSO transfected cells (Figure 4F–H). Similar results were obtained with U251MG cells transfected with 2′-MOE 2b-block SSO and treated with the same chemotherapies (Supplementary Figure S4A–C). These data suggest a link between *MKNK2* alternative splicing and the resistance of glioblastomas to chemotherapy and offer a possible solution for the unsuccessful treatment of these tumors.

### The 2b-block SSO inhibits glioblastoma formation *in vivo*

**Figure 5.**
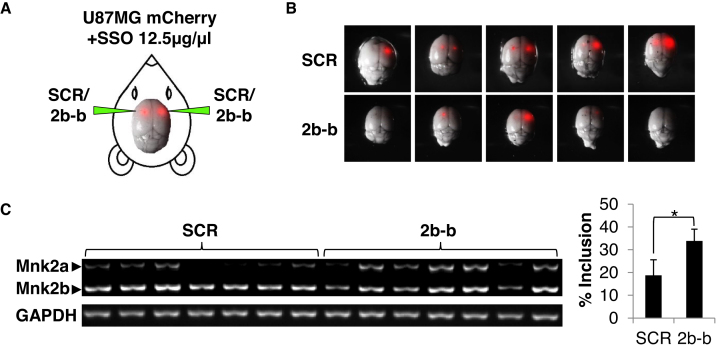
The 2b-block SSO inhibits glioblastoma formation *in vivo*. ( **A**) Schematic representation of injection of cells into the murine brain. (**B**) mCherry-labeled U87MG cells resuspended in PBS containing 2′-OMe 2b-block SSO or SCR oligo 12.5 μg/μl were injected intracranially into the striatum on both sides. After 14 days the mice were sacrificed and brains were photographed under a fluorescent dissecting microscope. (**C**) RT-PCR analysis of RNA isolated from the tumors described in (B). *P = 0.0087 two-sided, *n* = 7 for each group.

To examine if the 2b-block SSO can inhibit glioblastoma growth *in vivo*, we labeled U87MG cells with mCherry, in order to visualize the cells *in vivo* (Figure 5A). Cells were mixed with a solution of naked SSOs immediately before injection into the murine striatum of both brain hemispheres. Two weeks after injection the mice were sacrificed, the tumors were removed and visualized, and RNA was extracted. The mice injected with cells mixed with the 2b-block SSO developed notably smaller tumors, or no tumors at all, than the control group (Figure 5B). Analysis of RNA extracted from the tumors showed the expected splice switch; i.e. significantly higher Mnk2a mRNA levels in tumors containing 2b-block SSO treated cells than SCR–treated cells (Figure 5C). Although the present setting of the experiment is not a model for treatment *in vivo*, these results suggest that intracranially injected SSOs designed to modulate *MKNK2* alternative splicing can reduce glioblastoma tumor burden in mice.

## DISCUSSION

Here, we present the development of an SSO to modulate the splicing of *MKNK2* with potential to treat glioblastomas. Findings in recent years have demonstrated that the process of alternative splicing is deregulated in cancer and is involved in cancer initiation and progression. Drugs which target the general splicing process, such as Spliceostatin A, efficiently kill cancer cells, however they show high toxicity *in vivo* (18–20). In the past year, two SSOs which modulate-specific splicing events have been approved by the Food and Drug Administration (FDA). The first SSO for the treatment of Duchenne muscular dystrophy was developed by Serepta and the second, for the treatment of spinal muscular atrophy was developed by Dr Adrian Krainer's lab and Ionis pharmaceuticals (21,22). The success of these drugs encourages the development of splicing modulation for cancer therapy as well, a concept which has been proposed previously (23). In many cases alternative splicing can convert an oncogenic isoform into a tumor suppressor or *vice versa*. For example, many growth factor receptors such as EGFR, VEGFR, ERBB2, which are amplified in cancers, can also generate a soluble decoy receptor by alternative splicing (24). Furthermore, the short isoforms of BCL–X or MCL–1 induce apoptosis while their long counterparts are known to protect from apoptosis (25). The manipulation of the *BCL–X* pre–mRNA alternative splicing by 2′-OMe SSOs as an approach to induce apoptosis in cancer cells has been previously demonstrated (26).

Glioblastoma is a devastating disease with a median survival of ∼15 months (27). The current first line of care for brain cancer patients with poor prognosis is composed of surgical removal of the tumor, followed by chemotherapy and radiation treatments. Typically tumor excision is associated with a survival benefit. However, in addition to being risky and toxic, this course of action is non-specific and does not address the underlying biology of the tumor, which might play a crucial role in the development of the disease. Even though there have been attempts to develop targeted therapies for glioblastoma, such as EGFR and VEGFR inhibitors erlotinib (28) and tivozanib (29), to date there is no effective treatment for this cancer and development of drug resistance is one of the major problems with current therapies (30).

We previously found that *MKNK2* alternative splicing can generate a tumor suppressive isoform (Mnk2a) and an oncogenic isoform (Mnk2b; Figure 1A) (10). Moreover, Mnk2b is upregulated while Mnk2a is downregulated in many cancers, including glioblastoma (Figures 1 and 2; Supplementary Figures S1A and 2A) (10). We assume that due to variability between patients, the fact that these are not matched samples and low number of normal brain samples in the TCGA data, these results are an underestimation of the real splicing changes in glioblastoma tumors compared to normal tissue.

Here, we performed an oligonucleotide screen and identified the most efficient SSO that elevated Mnk2a levels in a dose-dependent manner (Figure 2) and inhibited brain cancer cell survival (Figure 3F and G; Supplementary Figures S2D-E and 3D-E). This effect is mediated through the phosphorylation of p38α and activation of the p38–MAPK stress response (Figure 3A–E; Supplementary Figure S2B and C), consistent with what is known (10). Furthermore, the significance of p38–MAPK signaling for the 2b-block SSO-mediated glioblastoma inhibition is demonstrated by the reversal of the signaling and biological effects promoted by the 2b-block SSO, using the p38–MAPK inhibitor SB203580. Cells treated with the SB203580 inhibitor following the transfection with the SSOs, exhibited weaker or no induction of p38α transcriptional targets, as well as increased survival and anchorage independent growth (Figure 4A–E). Given that we tested multiple SSOs that did not affect *MKNK2* splicing and did not inhibit the growth/survival of cancer cells, we are confident that this effect is specific. In addition, the identification of the biological pathway responsible for the anti-oncogenic effect, and its reversal, reinforce our conclusion that the 2b-block SSO is acting by directly targeting the *MKNK2* splicing.

Another characteristic of glioblastoma cells is their intrinsic resistance to conventional chemotherapy (31,32). Thus, we examined if *MKNK2* splicing modulation by the 2b-block SSO can sensitize glioblastoma cell lines to chemotherapeutic agents such as, CDDP, doxorubicin and TMZ. We found that the effect of the 2b-block SSO is amplified when combined with either CDDP, doxorubicin or TMZ, elevating the cytotoxicity of these chemotherapeutic agents up to 8-fold compared to each treatment alone (Figure 4F–H; Supplementary Figure S4A–C). While CDDP and doxorubicin are not currently used for treatment of glioblastoma, due to poor penetration of the blood brain barrier (33,34) and resistance mechanisms (35,36), we suggest that in combination with the 2b-block SSO and with direct delivery to the brain they could possibly prove beneficial. More relevant to glioblastoma treatment is the combination of the 2b-block SSO with TMZ, which is the current first line of chemotherapy for glioblastoma. These results suggests that the 2b-block SSO can significantly enhance the efficacy of current glioblastoma treatments.

At last, we sought to examine the ability of the 2b-block SSO to inhibit glioblastoma growth *in vivo*. The alternative splicing of the murine *Mknk2* mRNA does not occur in a mode similar to the human *MKNK2*, so the 2b-block SSO cannot be tested directly on mouse glioblastomas. Hence, it was necessary to perform xenograft experiments, using human glioblastoma cells, to test the activity of 2b-block *in vivo*. In order to assess the ability of the SSO to penetrate tumor cells *in vivo* without a transfection reagent and to provide a proof of concept for treatment of glioblastoma with SSOs *in vivo*, we co-injected the naked SSO together with mCherry–labeled U87MG cells intracranially and examined the tumors two weeks later. We found that the 2′-OMe phosphorothioated backbone 2b-block SSO both induced a shift in *MKNK2* splicing *in vivo* and inhibited tumor growth (Figure 5). These results suggest that modulation of *MKNK2* splicing by SSOs is a valid novel strategy to inhibit glioblastoma tumorigenesis. Taking into account its enhanced activity with chemotherapeutic drugs, *MKNK2* splicing modulation can be examined as a single or combined therapy for glioblastoma.

## DATA AVAILABILITY

Any non-commercial material presented in the manuscript can be obtained through an MTA.

## Supplementary Material

Supplementary Data

## Notes

**Disclaimer:** US granted patent number 2015-0141489-A1 pertains to the results presented in the paper.

## References

[1] Oltean S., Bates D.O. Hallmarks of alternative splicing in cancer. Oncogene. 2014; 33:5311–5318. 24336324 10.1038/onc.2013.533

[2] Dvinge H., Kim E., Abdel-Wahab O., Bradley R.K. RNA splicing factors as oncoproteins and tumour suppressors. Nat. Rev. Cancer. 2016; 16:413–430. 27282250 10.1038/nrc.2016.51 PMC5094465

[3] Karni R., de Stanchina E., Lowe S.W., Sinha R., Mu D., Krainer A.R. The gene encoding the splicing factor SF2/ASF is a proto-oncogene. Nat. Struct. Mol. Biol. 2007; 14:185–193. 17310252 10.1038/nsmb1209 PMC4595851

[4] Anczukow O., Rosenberg A.Z., Akerman M., Das S., Zhan L., Karni R., Muthuswamy S.K., Krainer A.R. The splicing factor SRSF1 regulates apoptosis and proliferation to promote mammary epithelial cell transformation. Nat. Struct. Mol. Biol. 2012; 19:220–228. 22245967 10.1038/nsmb.2207 PMC3272117

[5] Waskiewicz A.J., Flynn A., Proud C.G., Cooper J.A. Mitogen-activated protein kinases activate the serine/threonine kinases Mnk1 and Mnk2. EMBO J. 1997; 16:1909–1920. 9155017 10.1093/emboj/16.8.1909 PMC1169794

[6] Mamane Y., Petroulakis E., Rong L., Yoshida K., Ler L.W., Sonenberg N. eIF4E–from translation to transformation. Oncogene. 2004; 23:3172–3179. 15094766 10.1038/sj.onc.1207549

[7] Furic L., Rong L., Larsson O., Koumakpayi I.H., Yoshida K., Brueschke A., Petroulakis E., Robichaud N., Pollak M., Gaboury L.A. eIF4E phosphorylation promotes tumorigenesis and is associated with prostate cancer progression. PNAS. 2010; 107:14134–14139. 20679199 10.1073/pnas.1005320107 PMC2922605

[8] Ueda T., Sasaki M., Elia A.J., Chio I.I., Hamada K., Fukunaga R., Mak T.W. Combined deficiency for MAP kinase-interacting kinase 1 and 2 (Mnk1 and Mnk2) delays tumor development. PNAS. 2010; 107:13984–13990. 20679220 10.1073/pnas.1008136107 PMC2922567

[9] Scheper G.C., Parra J.L., Wilson M., Van Kollenburg B., Vertegaal A.C., Han Z.G., Proud C.G. The N and C termini of the splice variants of the human mitogen-activated protein kinase-interacting kinase Mnk2 determine activity and localization. Mol. Cell. Biol. 2003; 23:5692–5705. 12897141 10.1128/MCB.23.16.5692-5705.2003 PMC166352

[10] Maimon A., Mogilevsky M., Shilo A., Golan-Gerstl R., Obiedat A., Ben-Hur V., Lebenthal-Loinger I., Stein I., Reich R., Beenstock J. Mnk2 alternative splicing modulates the p38-MAPK pathway and impacts Ras-induced transformation. Cell Rep. 2014; 7:501–513. 24726367 10.1016/j.celrep.2014.03.041

[11] Dowdy S.F. Overcoming cellular barriers for RNA therapeutics. Nat. Biotechnol. 2017; 35:222–229. 28244992 10.1038/nbt.3802

[12] Ryan M., Wong W.C., Brown R., Akbani R., Su X., Broom B., Melott J., Weinstein J. TCGASpliceSeq a compendium of alternative mRNA splicing in cancer. Nucleic Acids Res. 2015; 44:D1018–D1022. 26602693 10.1093/nar/gkv1288 PMC4702910

[13] Venables J.P., Klinck R., Bramard A., Inkel L., Dufresne-Martin G., Koh C., Gervais-Bird J., Lapointe E., Froehlich U., Durand M. Identification of alternative splicing markers for breast cancer. Cancer Res. 2008; 68:9525–9531. 19010929 10.1158/0008-5472.CAN-08-1769

[14] Badger A.M., Bradbeer J.N., Votta B., Lee J.C., Adams J.L., Griswold D.E. Pharmacological profile of SB 203580, a selective inhibitor of cytokine suppressive binding protein/p38 kinase, in animal models of arthritis, bone resorption, endotoxin shock and immune function. J. Pharmacol. Exp. Ther. 1996; 279:1453–1461. 8968371

[15] Lee J.C., Kumar S., Griswold D.E., Underwood D.C., Votta B.J., Adams J.L. Inhibition of p38 MAP kinase as a therapeutic strategy. Immunopharmacology. 2000; 47:185–201. 10878289 10.1016/s0162-3109(00)00206-x

[16] Holohan C., Van Schaeybroeck S., Longley D.B., Johnston P.G. Cancer drug resistance: an evolving paradigm. Nat. Rev. Cancer. 2013; 13:714–726. 24060863 10.1038/nrc3599

[17] Siegfried Z., Karni R. The role of alternative splicing in cancer drug resistance. Curr. Opin. Genet. Dev. 2018; 48:16–21. 29080552 10.1016/j.gde.2017.10.001

[18] Kaida D., Motoyoshi H., Tashiro E., Nojima T., Hagiwara M., Ishigami K., Watanabe H., Kitahara T., Yoshida T., Nakajima H. Spliceostatin A targets SF3b and inhibits both splicing and nuclear retention of pre-mRNA. Nat. Chem. Biol. 2007; 3:576–583. 17643111 10.1038/nchembio.2007.18

[19] Eskens F.A., Ramos F.J., Burger H., O’Brien J.P., Piera A., de Jonge M.J., Mizui Y., Wiemer E.A., Carreras M.J., Baselga J. Phase I, pharmacokinetic and pharmacodynamic study of the first-in-class spliceosome inhibitor E7107 in patients with advanced solid tumors. Clin. Cancer Res. 2013; 19:6296–6304. 23983259 10.1158/1078-0432.CCR-13-0485

[20] Dehm S.M. Test-firing ammunition for spliceosome inhibition in cancer. Clin. Cancer Res. 2013; 19:6064–6066. 24097858 10.1158/1078-0432.CCR-13-2461 PMC3839097

[21] Voit T., Topaloglu H., Straub V., Muntoni F., Deconinck N., Campion G., De Kimpe S.J., Eagle M., Guglieri M., Hood S. Safety and efficacy of drisapersen for the treatment of Duchenne muscular dystrophy (DEMAND II): an exploratory, randomised, placebo-controlled phase 2 study. Lancet Neurol. 2014; 13:987–996. 25209738 10.1016/S1474-4422(14)70195-4

[22] Finkel R.S., Chiriboga C.A., Vajsar J., Day J.W., Montes J., De Vivo D.C., Yamashita M., Rigo F., Hung G., Schneider E. Treatment of infantile-onset spinal muscular atrophy with nusinersen: a phase 2, open-label, dose-escalation study. Lancet. 2016; 388:3017–3026. 27939059 10.1016/S0140-6736(16)31408-8

[23] Sazani P., Kole R. Therapeutic potential of antisense oligonucleotides as modulators of alternative splicing. J. Clin. Invest. 2003; 112:481–486. 12925686 10.1172/JCI19547 PMC171400

[24] Vorlová S., Rocco G., LeFave C.V., Jodelka F.M., Hess K., Hastings M.L., Henke E., Cartegni L. Induction of antagonistic soluble decoy receptor tyrosine kinases by intronic polyA activation. Mol. Cell. 2011; 43:927–939. 21925381 10.1016/j.molcel.2011.08.009 PMC3781938

[25] Akgul C., Moulding D., Edwards S. Alternative splicing of Bcl-2-related genes: functional consequences and potential therapeutic applications. Cell. Mol. Life Sci. 2004; 61:2189–2199. 15338051 10.1007/s00018-004-4001-7 PMC11138917

[26] Mercatante D.R., Mohler J.L., Kole R. Cellular response to an antisense-mediated shift of Bcl-x pre-mRNA splicing and antineoplastic agents. J. Biol. Chem. 2002; 277:49374–49382. 12381725 10.1074/jbc.M209236200

[27] Young R.M., Jamshidi A., Davis G., Sherman J.H. Current trends in the surgical management and treatment of adult glioblastoma. Ann.Transl. Med. 2015; 3:121–136. 26207249 10.3978/j.issn.2305-5839.2015.05.10 PMC4481356

[28] Sathornsumetee S., Desjardins A., Vredenburgh J.J., McLendon R.E., Marcello J., Herndon J.E., Mathe A., Hamilton M., Rich J.N., Norfleet J.A. Phase II trial of bevacizumab and erlotinib in patients with recurrent malignant glioma. Neuro Oncol. 2010; 12:1300–1310. 20716591 10.1093/neuonc/noq099 PMC3018944

[29] Kalpathy-Cramer J., Chandra V., Da X., Ou Y., Emblem K.E., Muzikansky A., Cai X., Douw L., Evans J.G., Dietrich J. Phase II study of tivozanib, an oral VEGFR inhibitor, in patients with recurrent glioblastoma. J. Neurooncol. 2017; 131:603–610. 27853960 10.1007/s11060-016-2332-5 PMC7672995

[30] Messaoudi K., Clavreul A., Lagarce F. Toward an effective strategy in glioblastoma treatment. Part I: resistance mechanisms and strategies to overcome resistance of glioblastoma to temozolomide. Drug Discov. Today. 2015; 20:899–905. 25744176 10.1016/j.drudis.2015.02.011

[31] Sarkaria J.N., Kitange G.J., James C.D., Plummer R., Calvert H., Weller M., Wick W. Mechanisms of chemoresistance to alkylating agents in malignant glioma. Clin. Cancer Res. 2008; 14:2900–2908. 18483356 10.1158/1078-0432.CCR-07-1719 PMC2430468

[32] Benhar M., Engelberg D., Levitzki A. Cisplatin-induced activation of the EGF receptor. Oncogene. 2002; 21:8723–8731. 12483525 10.1038/sj.onc.1205980

[33] Jacobs S.S., Fox E., Dennie C., Morgan L.B., McCully C.L., Balis F.M. Plasma and cerebrospinal fluid pharmacokinetics of intravenous oxaliplatin, cisplatin, and carboplatin in nonhuman primates. Clin. Cancer Res. 2005; 11:1669–1674. 15746072 10.1158/1078-0432.CCR-04-1807

[34] Ohnishi T., Tamai I., Sakanaka K., Sakata A., Yamashima T., Yamashita J., Tsuji A. In vivo and in vitro evidence for ATP-dependency of P-glycoprotein-mediated efflux of doxorubicin at the blood-brain barrier. Biochem. Pharmacol. 1995; 49:1541–1544. 7763297 10.1016/0006-2952(95)00082-b

[35] Han J., Jun Y., Kim S.H., Hoang H.H., Jung Y., Kim S., Kim J., Austin R.H., Lee S., Park S. Rapid emergence and mechanisms of resistance by U87 glioblastoma cells to doxorubicin in an in vitro tumor microfluidic ecology. PNAS. 2016; 113:14283–14288. 27911816 10.1073/pnas.1614898113 PMC5167174

[36] Nagane M., Levitzki A., Gazit A., Cavenee W.K., Huang H.J. Drug resistance of human glioblastoma cells conferred by a tumor-specific mutant epidermal growth factor receptor through modulation of Bcl-XL and caspase-3-like proteases. PNAS. 1998; 95:5724–5729. 9576951 10.1073/pnas.95.10.5724 PMC20446

