# Supplementary material for: Modulation of MKNK2 alternative splicing by splice-switching oligonucleotides as a novel approach for glioblastoma treatment
Source: Nucleic Acids Res. 2018 Oct 17;46(21):11396–404. doi: 10.1093/nar/gky921 (PMC6265459; doi:10.1093/nar/gky921)
Supplement: Supplementary Data [file gky921_supplemental_files.pdf]

Supplemental Material

Figure S1. The 2b–block SSO promotes Mnk2a production and activates the p38–MAPK stress pathway in hepatocellular carcinoma and metastatic breast carcinoma cells.

A. RT-PCR analysis of mRNA isolated from either HuH7 hepatocellular carcinoma (left panel) or MDA–MB–231 metastatic breast carcinoma cells (right panel) transfected with 2.5μM of either SCR, 2a-block or 2b-block 2'-OMe oligos 72h post transfection. *p<0.01, n=6 for each group. **B.** Western blot analysis of protein lysates from either HuH7 hepatocellular carcinoma (left panel) or MDA–MB–231 metastatic breast carcinoma cells (right panel) transfected with 2.5μM of either SCR, 2a-block or 2b-block 2'-OMe oligos 72h post transfection. Membranes were probed with the indicated antibodies. **C,D.** qRT- PCR analysis of mRNA isolated from either HuH7 hepatocellular carcinoma (C) or MDA–MB–231 metastatic breast carcinoma cells (D) transfected with 2.5μM of either SCR, 2a-block or 2b-block 2'-OMe oligos 72h post transfection. *p<0.05 two–sided, **p<0.01 two–sided, n=3 for each group.

Figure S2. The 2b-block SSO switches *MKNK2* alternative splicing, activates the p38α–MAPK stress pathway and inhibits the oncogenic properties of U251MG glioblastoma cells.

A. RT-PCR analysis of mRNA isolated from U251MG glioblastoma cells transfected with 1μM of the indicated 2'-MOE oligos 72h post transfection. **B.** Western blot analysis of protein lysates from U251MG glioblastoma cells transfected with 1μM of the indicated 2'-MOE oligos 72h post transfection. Membranes were probed with the indicated antibodies. **C.** qRT-PCR analysis of mRNA isolated from U251MG glioblastoma cells transfected with 1μM of the indicated 2'-MOE oligos 72h post transfection. *p<0.01 two–sided, n=3. **D.** Colony formation in soft agar assay performed on U251MG glioblastoma cells transfected with 1μM of the indicated 2'-MOE oligos. n=3 wells for each group, in each well 10 fields were counted. **E.** Clonogenic assay performed on U251MG glioblastoma cells transfected with 1μM of the indicated 2'-MOE oligos and seeded at low density. The number of colonies in each treatment was normalized to number of colonies of non-treated cells. n=3 wells for each group.

Figure S3. SSO mediated switch in Mnk2 splicing affects survival of hepatocellular carcinoma and metastatic breast carcinoma cells.

A. Colony formation in soft agar assay performed on MDA-MB-231 cells transfected with 2.5µM of the indicated 2'-OMe oligos. **p<0.01 two–sided, n=3 wells for each group, in each well 10 fields were counted. **B, C.** Clonogenic assay performed on HuH7 (B) and MDA-MB-231 (C) cells transfected with 2.5µM of the indicated 2'-OMe oligos and seeded at a low density. The number of colonies in each treatment was normalized to number of colonies in cells treated with SCR oligo. *p<0.05 two–sided, **p<0.01 two–sided, n=3 wells for each group. **D.** Colony formation in soft agar assay performed on U87MG glioma cells transfected with the indicated 2'-MOE oligos. **p<0.01 two–sided, n=2 wells for each group, in each well 10 fields were counted. **E.** Clonogenic assay performed on U87MG glioma cells transfected with the indicated 2'-MOE oligos and seeded at a low density. The number of colonies in each treatment was normalized to number of colonies in cells treated with SCR oligo.

Figure S4. The 2b–block 2'-MOE SSO sensitizes U87MG and U251MG glioblastoma cells to TMZ, doxorubicin and CDDP in culture.

A. Trypan blue exclusion assay performed on U251MG cells transfected with 1µM of the indicated 2'-MOE oligos and treated with 8µg/µl doxorubicin (blue bars) or vehicle (black bars) for 48h. *p≤0.05 two–sided, n=3 wells for each group. **B.** Trypan blue exclusion assay performed on U251MG cells transfected with 1µM of the indicated 2'-MOE oligos and treated with 100µM CDDP (blue bars) or vehicle (black bars) for 48h. *p≤0.05 two–sided, n=3 wells for each group. **C.** Trypan blue exclusion assay performed on U251MG cells transfected with 1µM of the indicated 2'-MOE oligos and treated with 500µM TMZ (blue bars) or vehicle (black bars) for 72h. *p≤0.05 two–sided, **p≤0.01 two–sided, n=3 wells for each group.

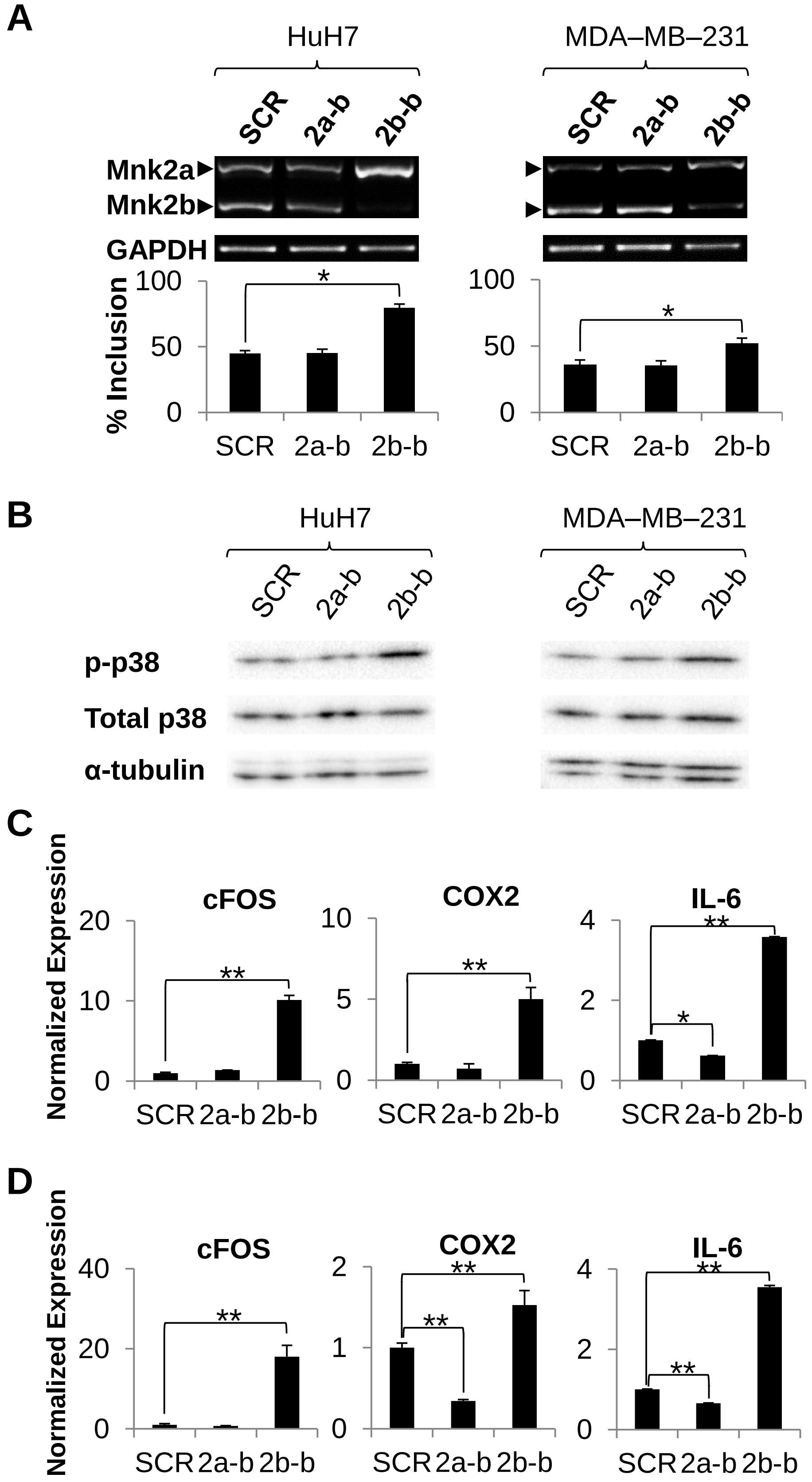

Mogilevsky et al., Figure S1

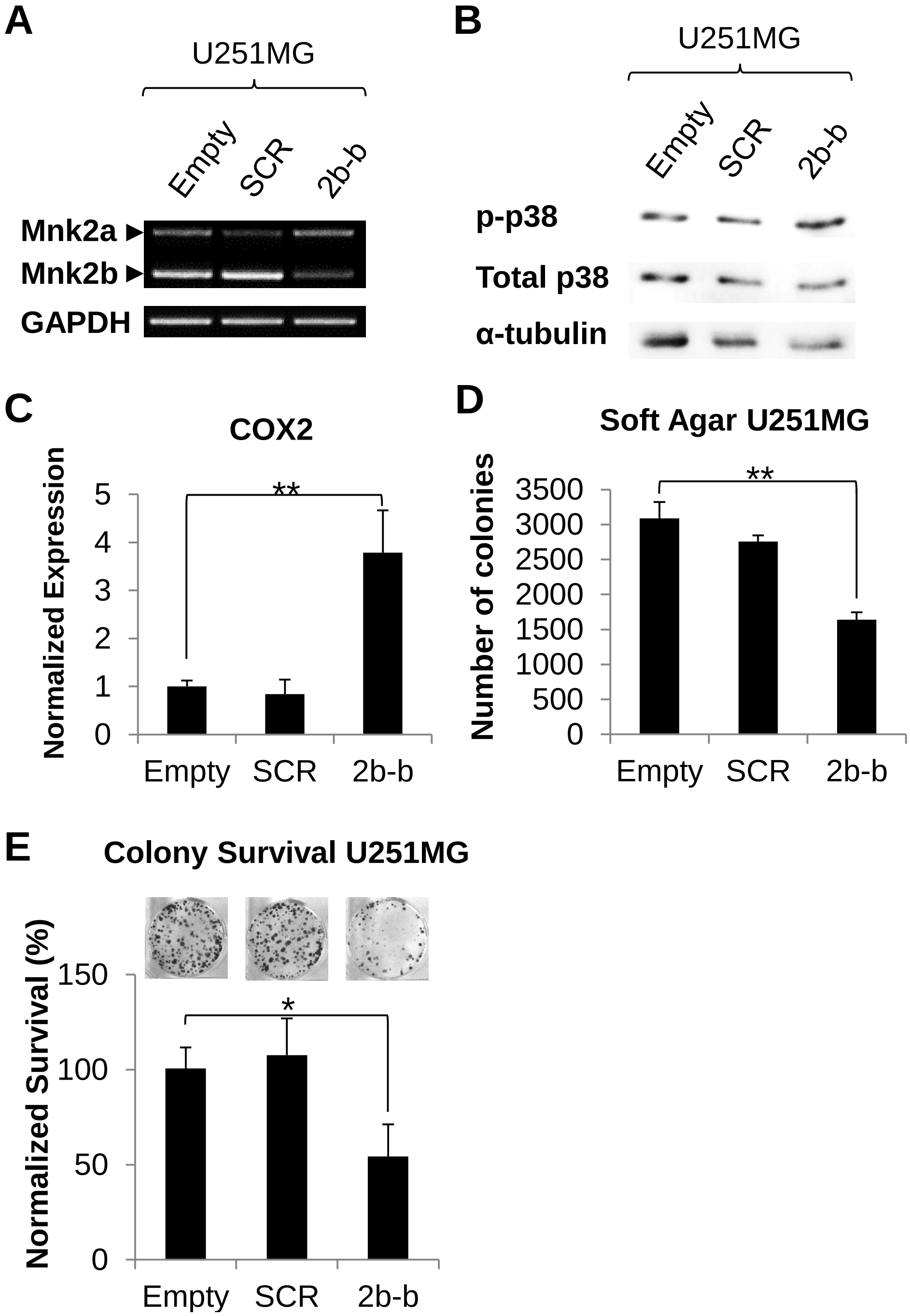

Mogilevsky et al., Figure S2

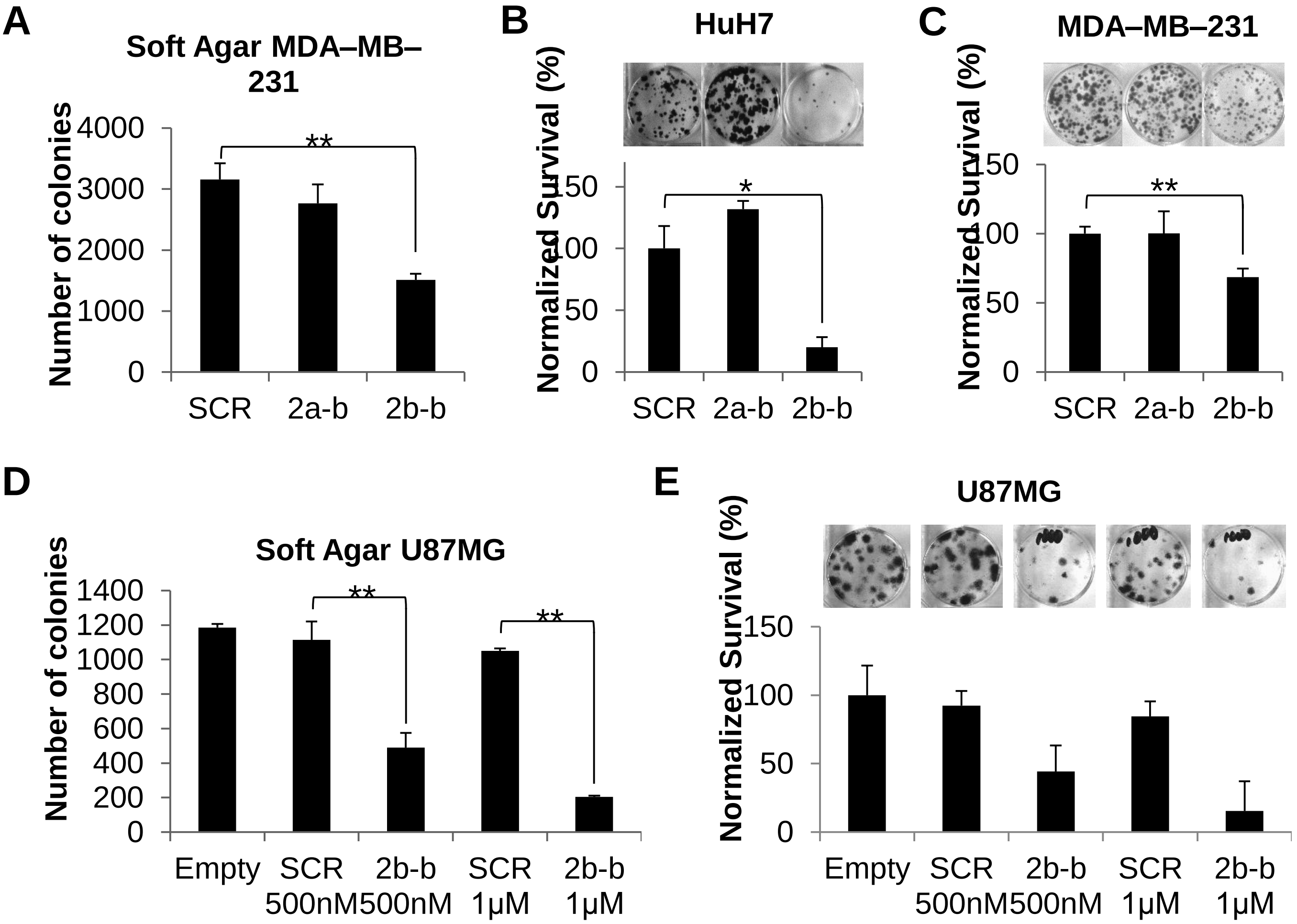

Mogilevsky et al., Figure S3

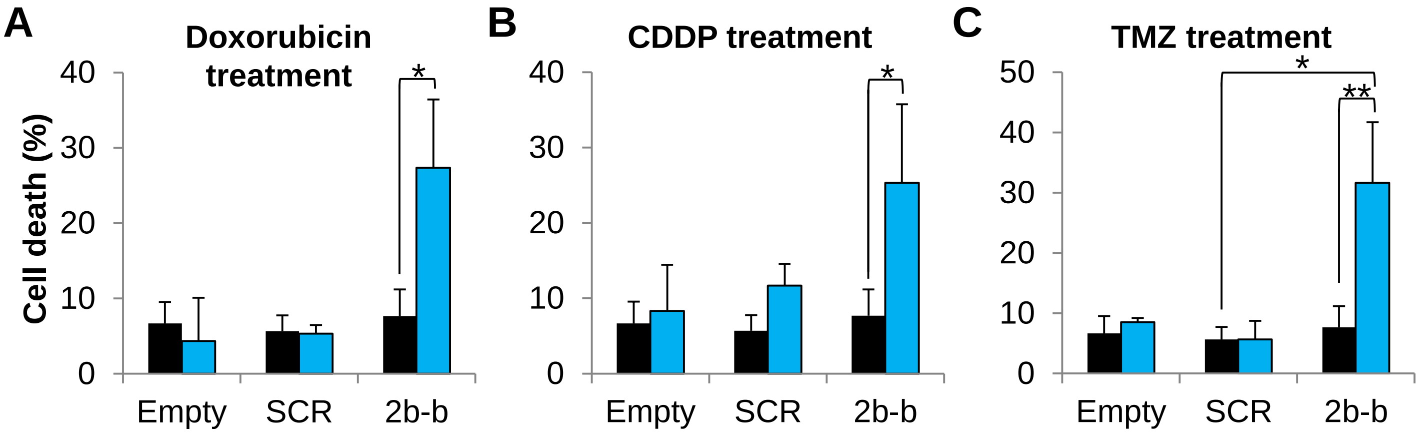

Mogilevsky et al., Figure S4
